# Aging Population and Lacking Sanitation Governance: Global Challenges in Alleviating Deaths from Unsafe Rural Sanitation

**DOI:** 10.1021/envhealth.4c00246

**Published:** 2025-02-28

**Authors:** Zixuan Wang, Pengyu Li, Wenkai Li, Yingnan Cao, Jianguo Liu, Lin Li, Junxin Liu, Tianlong Zheng

**Affiliations:** a State Key Laboratory of Environmental Aquatic Chemistry, Research Center for Eco-Environmental Sciences, Chinese Academy of Sciences, Beijing 100085, China; b University of Chinese Academy of Sciences, Beijing 100049, China; c National Joint Research Center for Ecological Conservation and High Quality Development of the Yellow River Basin, Beijing 100012, China; d SCEGC No. 12 Construction Engineering Group Co., Ltd., Ankang National High-Tech Industries Development Zone, Ankang 725000, Shaanxi China; e Key Laboratory of Environmental Pollution Control and Remediation at Universities of Inner Mongolia Autonomous Region, College of Resources and Environmental Engineering, 74507 Inner Mongolia University of Technology, Hohhot 010051, Inner Mongolia China

**Keywords:** Rural aera, Global burden of disease, Unsafe sanitation, Environmental risk, Prediction, Decomposition analysis

## Abstract

With the rapid pace of global urbanization, health risks faced by rural communities are often overlooked. Deaths Attributable to Unsafe Sanitation in Rural areas (DAUSRs) are influenced by demographic factors, disease mortality rates, and environmental sanitation conditions. However, most studies have been limited in scope and scale and lack a comprehensive evaluation framework for global DAUSRs. Therefore, this study estimated the global DAUSRs from 2000 to 2030, using data from the Global Burden of Disease (GBD) and the World Health Organization (WHO). We employed methods such as comparable risk assessment, Bayesian age (period) models, and AutoRegressive Integrated Moving Average (ARIMA) models. Changes in the DAUSRs and their influencing factors were evaluated by applying a decomposition method to assess the impact of population dynamics, sanitation conditions, age structure, and disease mortality rates. The results indicated that despite improvements in rural sanitation, 12.2% of rural populations will still lack access to sanitary toilets in 2030, with an estimated 243,000 deaths (CI: 147,000–441,000) due to unsafe rural sanitation environments. This outcome highlights the need for better rural sanitation governance to provide for demographic shifts, such as aging and declining fertility rates, which are key drivers of DAUSRs. Regions such as Africa and Southeast Asia are at a higher risk with higher diarrhea-related mortality rates in rural areas. We suggest comprehensive measures, including enhancing rural medical facilities, improving sanitation infrastructure, and focusing on vulnerable groups, such as the elderly and children. These measures could inform global rural environmental and public health policies.

## Graphical abstract

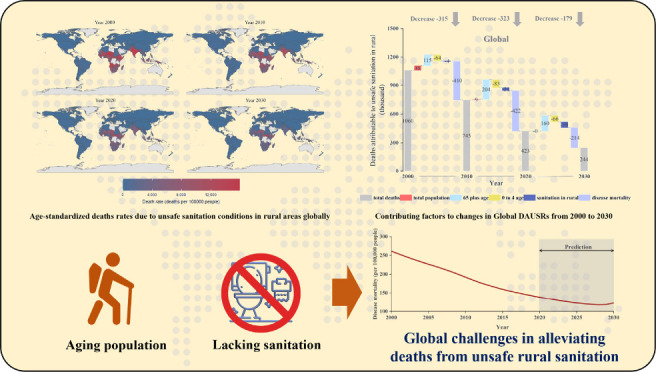

## 1 Introduction

Deaths Attributable to Unsafe Sanitation in Rural areas (DAUSRs) are influenced by demographic factors, disease mortality rates, and environmental sanitation conditions.^1^ Exposure to unsafe sanitation practices, such as open defecation and inadequate sewage infrastructure, are a primary contributor to the global burden of certain diseases, particularly diarrhea. According to the World Health Organization (WHO), unsafe sanitation conditions contribute to 564,000 deaths worldwide each year (https://www.who.int/zh/news-room/fact-sheets/detail/sanitation). Due to DAUSRs the United Nations Sustainable Development Goal 3, particularly target 3.9, aims to significantly reduce deaths and illnesses from environmental and occupational risks and pollution by 2030. However, with the rapid pace of global urbanization, the health risks faced by rural communities are often overlooked, which has led to a significant skew in public health policies and resource allocation in urban areas. Rural regions encountering severe environmental health challenges have increased disease burden, especially in low-income countries or regions lacking basic sanitary facilities.^2^ Addressing these issues is crucial to achieving equitable health outcomes and foster sustainable development.

The issue of unsafe environmental health conditions in rural areas worldwide has increasingly captured the attention of nations, regions, and organizations. In response to the heightened health risks disproportionately affecting rural communities, a variety of interventions have been implemented to alleviate disease burden. Several countries, including China, Brazil, India, Kenya, and Bangladesh, have taken proactive measures to address these issues. These efforts have encompassed a wide range of strategies, such as strengthening infrastructure to improve access to clean water and sanitation facilities, promoting sustainable agricultural practices to reduce environmental pollution, establishing stringent health regulations to ensure compliance with safety standards, and intensifying public health awareness campaigns to educate communities about healthy behaviors and practices.^3–6^

At the regional level, collaborative efforts are essential to addressing shared environmental health challenges. These collaborations have emphasized cross-border water resource management to ensure the sustainable use and protection of transboundary water bodies, as well as joint environmental monitoring to track and respond to environmental changes that may impact public health. Such approaches are critical for maintaining the safety and integrity of shared natural resources, which are often vital for the survival and wellbeing of rural communities.^7^ In addition, international organizations have played a crucial role in supporting these efforts by providing technical expertise, financial assistance, and capacity-building initiatives. These organizations have facilitated the transfer of knowledge and skills, enabling local communities to better manage rural environmental health challenges and enhance resilience to future risks. By bolstering local abilities and fostering sustainable development, these efforts have contributed to improving overall health and quality of life in rural areas.^8^ Although notable advancements have been made through various interventions to address rural environmental health issues, a comprehensive understanding of the efficacy of these efforts remains elusive. Hence, there is an urgent need for further rigorous investigation to assess their long-term implications, pinpoint potential shortcomings and obstacles, and devise more effective strategies tailored to address environmental health concerns specific to rural settings.

Most quantitative studies on environmental-exposure-induced death have focused on air pollution. For instance, Xu et al. utilized the comparable risk assessment framework of the Global Burden of Disease (GBD), to estimate the number of deaths attributable to PM2.5 air pollution in China, using different Shared Socioeconomic Pathways (SSPs) and representative concentration pathway (RCPs) scenarios, between 2000 and 2035.^9^ Second, previous research has predominantly focused on the combined effects of global drinking water quality, sanitation facilities, and personal hygiene on diarrhea prevalence, without separately analyzing the disease burden associated with environmental sanitation conditions.^10–13^ Typically, these studies have examined the impact of various environmental stressors, such as water pollution, economic factors, and precipitation, individually, on diseases rather than within an integrated framework that considers the interactions among these factors. Consequently, the relative contribution of each factor to mortality was not adequately differentiated. Furthermore, researchers have rarely focused on deaths caused by unsafe rural environmental sanitation.^14^ Although some studies have attempted to quantify the impact of unsafe environmental health on diseases, most have been limited in scope and scale and lack a comprehensive evaluation framework for global DAUSRs. There is an urgent need to better understand the spatiotemporal patterns of global DAUSRs and the relative contributions of their driving factors.

This study aimed to comprehensively estimate the DAUSRs from 2000 to 2030. We first estimated the global DAUSRs for the period 2000–2020 by integrating epidemiological models from the GBD in 2021 with environmental health data from the WHO. Subsequently, we utilized Bayesian age-period-cohort (BAPC) models and AutoRegressive Integrated Moving Average (ARIMA) models to forecast disease mortality rates and rural sanitation conditions, allowing us to estimate the global DAUSRs from 2020 to 2030, and analyze the changes in DAUSRs and its driving factors. We then employed a decomposition approach to evaluate the relative contributions of population dynamics, sanitation conditions, age structure, and changes in disease mortality rates to DAUSRs.

## 2 Materials and Methods

This study integrates Comparable Risk Assessment (CRA), BAPC models, ARIMA models, and decomposition analysis into a comprehensive modeling framework (Figure 1).

**Figure 1:**
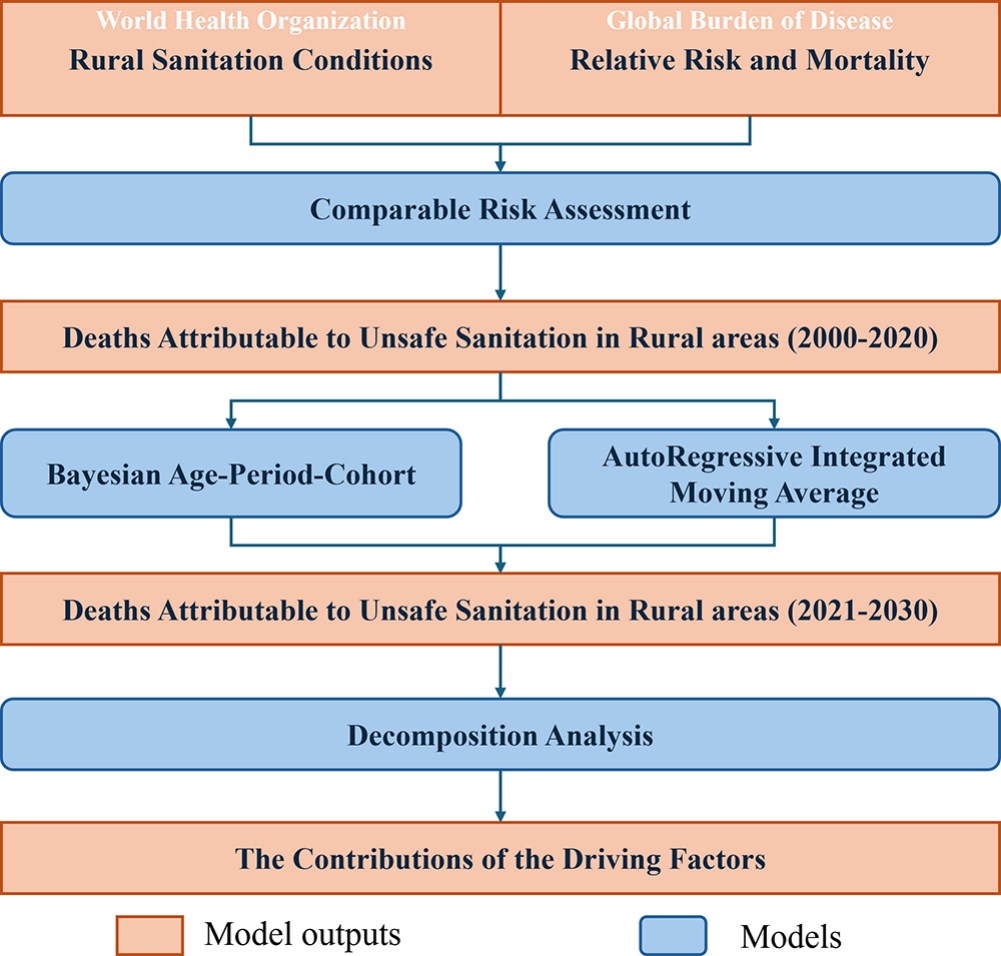
Modeling framework for the deaths attributable to unsafe sanitation in rural areas (DAUSRs).

### 2.1 Overview of the Study Area

We assessed deaths attributed to unsafe sanitation in rural areas across 154 countries by employing 2 classification methods: Case 1 categorizes the countries into Africa, the Mediterranean, Europe, the Americas, Southeast Asia, and the Western Pacific; Case 2 sorts them into high-, upper-middle-, lower-middle-, and low-income countries according to the WHO classification (https://datahelpdesk.worldbank.org/knowledgebase/articles/378834-how-does-the-world-bank-classify-countries). The temporal scale spans from 2000 to 2030, with projections from 2021 to forecast the mortality rate and the number of age-standardized deaths due to unsafe sanitation facilities by 2030.

### 2.2 Indicator Selection and Data Sources

The data used in this study included those on sanitation facilities, disease mortality, historical socioeconomic projections, future societal projections, and additional geographical information (Table 1).

**Table 1:** Data Structure and Sources^a^

Data		Sources
Historical data (2010–2020)	Diarrhea mortality, RR	GBD 2021 (https://vizhub.healthdata.org/gbd-results/)
	Age structure	
	SDI	
	Disease mortality	
	Rural population	WHO (https://www.who.int/data/inequality-monitor/data)
	Unsafe sanitation	
Future data (2021–2030)	Population	WHO (https://population.un.org/wpp/Download/Standard/MostUsed/)
	Age structure	

a GBD: Global Burden of Disease; RR: relative risk; SDI: Sociodemographic Index; WHO: World Health Organization.

We retrieved mortality rates from diarrhea, relative risks (RR), and Sociodemographic Index (SDI) data from 2000 to 2020 for 204 countries globally from the GBD 2021 database. We analyzed deaths from diarrhea due to unsafe sanitation facilities (https://ghdx.healthdata.org/). The SDI, a composite indicator of regional development based on the average educational attainment of the population aged 15 and above, the total fertility rate for those under 25 years, and per capita income, was set as a fixed effect in the linear regression because of its proven significance as a predictive factor.^15^ Data on the global rural population and sanitation conditions were obtained from the WHO, which reported on rural sanitation facilities across 170 countries from 2000 to 2021 (https://www.who.int/data/inequality-monitor/data). The research involved preprocessing multisource data, excluding countries with outlier data and missing data issues. Ultimately, the DAUSRs were estimated for 154 countries.

Exposure to unsafe sanitation was defined based on the type of toilet used in rural households. Sanitation facilities were categorized into three distinct types: unimproved, improved, and those connected to sewerage or septic systems, as established by the Joint Monitoring Program for Water Supply, Sanitation, and Hygiene, a collaboration between the WHO and the United Nations International Children’s Emergency Fund (UNICEF) (https://www.unicef.org/zh). Improved sanitation facilities included improved ventilated pit latrines, composting toilets, and pit latrines with slabs, while unimproved facilities included open defecation sites, latrines lacking proper structures, and systems discharging into streams or open fields. Toilets connected to sewerage systems consisted of flush toilets or toilets linked to a sewer or septic system.

Future socioeconomic data encompassed national-level figures on the global population and age structure.

### 2.3 Analytical Methods

#### 2.3.1 Comparable Risk Assessment

The Conceptual Framework of CRA was developed by Brauer et al.^16^ It establishes a causal network of risks or causes that are hierarchically organized concerning health outcomes. This framework facilitates the quantification of risks or causes at any level within a structure. Moreover, when calculating the population attributable fraction (PAF) for varying rural sanitation conditions, it is essential to employ a multifactorial approach to ensure the reliability of the results.^16^ The number of deaths was derived using equations 1–3):

$${\mathrm{DAUSR}}_{t}=\underset{a,d}{\sum }\left({\mathrm{POP}}_{t}\times {\mathrm{AgeP}}_{a,t}\times {\mathrm{Rate}}_{a,d,t}\times {\mathrm{PAF}}_{a,d,t}\right)$$ 1

where POP*_t_* is the total population in year *t*, AgeP_*a*_ *,*_*t*_ is the proportion of the population aged *a* in year *t*, Rate*_a,d,t_* represents the mortality rate for disease type *d* in the population within age group *a* during year *t*, and PAF*_a,d,t_* refers to the population-attributable fraction for disease type *d* in the population within age group *a* during year *t*;

$$\mathrm{PA}F_{A,i}=\frac{\int_{C}p_{A}\left(RR_{A,i}-1\right)dC}{\int_{C}p_{A}\left(RR_{A,i}-1\right)dC+1}=\frac{R{\mathrm{R̅}}_{A,i}-1}{R{\mathrm{R̅}}_{A,i}}$$ 2

where RR_*A,i*_ denotes the relative risk of disease *i* attributable to environmental factor *A* and *p*_*A*_ represents the proportion of the target population exposed to pollution or other risk factors.

$$\mathrm{PAF}=1-\underset{A}{\prod }\left(1-\mathrm{PA}F_{A,i}\right)$$ 3

The multifactorial approach takes into account the combined effects of multiple risk factors. By calculating the PAF for each individual risk factor and multiplying them together (for independent risk factors), we can obtain the overall PAF that considers all of the risk factors. This approach allows us to assess the contribution of individual risk factors to the disease burden, while holding other risk factors constant.

#### 2.3.2 Health Penetration Rate Estimation

In this study, the distribution of the three distinct population categories was normalized into two indices: the prevalence of the total population utilizing sewer or septic tank systems and the fraction of individuals employing enhanced sanitation infrastructure within the subpopulation not equipped with sewer or septic tank access.^17^ This is delineated in equations 4–6:

$$\mathrm{sewer}=\frac{\mathrm{persons with sewer or septic connection}}{\mathrm{persons with nonmissing response}}$$ 4

$$\mathrm{improved}=\left(\frac{\mathrm{persons using improved facilities}}{\mathrm{persons without sewer or septic connection}}\right)\left(1-\mathrm{sewer}\right)$$ 5

unimproved=1−(sewer+improved) 6

#### 2.3.3 BAPC Modeling

In this study, we applied the BAPC model using a Bayesian framework that allowed for the incorporation of prior knowledge and provided a probabilistic measure of uncertainty in the estimates. The model was fitted by using the Markov Chain Monte Carlo (MCMC) method to estimate the posterior distribution of the model parameters. The BAPC model is a statistical approach used to analyze and predict mortality rates while accounting for the effects of age, period, and cohort size.^18^ This approach is particularly valuable in epidemiological research, where trends in disease rates across birth cohorts, age groups, and time periods must be elucidated. The principles of the model are as follows:

- Age effects (*A*) are the changes in mortality rates as individuals age.
- Period effects (*P*) capture the changes in mortality rates that occur over time and simultaneously affect all age groups.
- Cohort effects (*C*) reflect changes in mortality rates associated with different birth cohorts and are expressed as the interaction between the age and period.

The BAPC model can be mathematically represented as

$$\log\left(\mu_{ijk}\right)=\alpha +A_{i}+P_{j}+C_{k}+\epsilon_{ijk}$$ 7

where *μ*_*ijk*_ is the mortality rate corresponding to age group *i*, period *j*, and cohort *k*, α is the baseline mortality rate at the reference point, *A*_*i*_ is the age-specific effect for age group *i* as indicated by the term, *P*_*j*_ denotes the period-specific effect for time period *j*. Additionally, *C*_*k*_ represents the cohort effect specific to individuals born in cohort *k*. The random error term is designated as *ϵ*_*ijk*_.

#### 2.3.4 ARIMA Modeling

Using Foreman et al.,^19^ we employed the ARIMA model to forecast rural sanitation conditions for the period 2021–2030. The ARIMA model, widely utilized in demographic and disease epidemiological trends,^20^ predicts patterns by analyzing the time series of historical data, with the series being rendered smooth through differencing to construct the ARIMA model. The ARIMA model is shown in equation 8:

$$\left(1-\overset{p}{\underset{i=1}{\sum }}\varphi_{i}L^{i}\right){\left(1-L\right)}^{d}X_{t}=\left(1+\overset{q}{\underset{j=1}{\sum }}\theta_{j}L^{J}\right)\epsilon_{t}$$ 8

where *φ*_*i*_ is the influence of past values of a time series on its current value, *L* is the lag operator, where *L* ^*i*^ *X*_*t*_ is the value of the time series at time *i*, *d* is the degree of differencing required to transform a nonstationary time series into a stationary one; *θ*_*j*_ is the moving average (MA) parameter, reflecting the impact of the error term of the time series on its present value; *ϵ*_*t*_ denotes the residual unaccounted for by the model.

Ultimately, using the estimates of sanitation coverage and disease-specific mortality rates alongside the population, this study employed a Comparable Risk Assessment framework model (as shown in equation 1) to quantify the DAUSRs from 2020 to 2030. Furthermore, the lower and upper bounds of the 95% confidence intervals for the mortality population were determined using the confidence interval (CI) limits of sanitation coverage and disease-specific mortality rates.

#### 2.3.5 Decomposition Analysis

Building on the studies of Cohen et al. and Yue et al.,^21,22^ we employed a decomposition technique to quantify the effects of age structure, population size, prevalence of rural sanitation facilities, and associated disease mortality rates on disease-related fatalities. In this decomposition analysis, we incrementally introduced variations in the age structure, population, rural sanitation facility prevalence, and disease mortality rates to calculate the number of deaths attributable to unsafe rural sanitation facilities, ultimately obtaining the contribution of each factor to the DAUSRs. To ensure the robustness of our findings, we considered all possible sequences in which these factors could be introduced and computed the average contribution to account for any potential ordering effects, ensuring that the total effects of all driving factors equal the change in DAUSRs. The decomposition method simplified the nonlinear relationships between these four driving factors and the changes in disease-related mortality into direct linear associations. By considering the interplay among these factors, this approach ensured that the quantified deaths due to unsafe rural sanitation facilities accurately reflected the contributions of the driving factors.^21^

### 2.4 Statistical Method

We employed the BAPC model to predict disease mortality rates using R Language V4.4.2 and the INLA package (https://rdrr.io/rforge/BAPC/). World map visualizations were generated using the “rnaturalearth” package in R. Additionally, time series forecasting of rural sanitation conditions across various countries was conducted using the “pmdarima” package in Python (https://alkaline-ml.com/pmdarima/). We decomposed and analyzed global DAUSRs from 2000 to 2030 using Python.

## 3 Results and Discussion

### 3.1 Trends in Driving Factors

The prediction performance, based on both the BAPC and ARIMA models, is presented in Supporting Information, Table S1. From 2000 to 2030, the global rural sanitation usage rates for “Improved Settings” and “Safely Managed” increased by 11.9% and 6.45%, respectively. Concurrently, the “Unimproved & Untreated Settings” decreased by 18.4% (Figure 2a). The projected trend of global rural “Unsafe sanitation” usage suggests that while regions and countries are making varying levels of progress in managing rural environmental sanitation, an estimated 12.2% of rural populations will lack access to sanitary toilets and will continue to practice open defecation in 2030. In the other two scenarios, the proportion of the rural population with “Improved Settings” sanitation is expected to rise to 64.8%, while that with “Safely Managed” sanitation will remain at only 23.0%, indicating that there is significant room for improvement. Our findings revealed a slow growth in the proportion of “Safely Managed” rural sanitation facilities. This result is similar to the 2030 projection of the SDG, which suggests that without accelerated improvement, the goal of eliminating open defecation, as stated in SDG 6.2, will remain unachieved by 2030 (https://www.un.org/sustainabledevelopment/zh/water-and-sanitation/).

**Figure 2:**
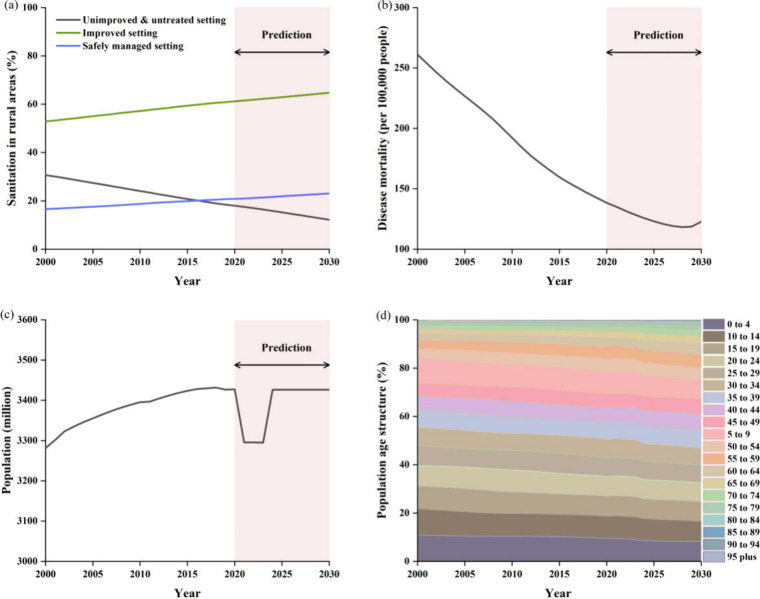
Trends of influencing factors from 2000 to 2030. (a) Changes in the proportions of three types of rural sanitation conditions globally; (b) Changes in global diarrheal mortality rates; (c) Changes in the global rural population; (d) Changes in the age structure of rural residents worldwide.

Diarrhea caused by “unsafe sanitation” corresponds to a significant disease burden. With the combined effects of socioeconomic development and improvements in healthcare, the model showed a notable decrease in the global mortality rate due to diarrhea between 2000 and 2030 (Figure 2b). Similarly, mortality rates across different age groups exhibited a declining trend. However, it is worth noting that the mortality rate for diarrhea remains high among individuals aged over 65 years and children aged 0–4, indicating that these populations will continue to be at significant risk (Figure S1). This predicted result contrasts with an estimation made by the WHO, that global childhood diarrheal mortality will reduce to 1 per 1000 by 2025. This discrepancy may be attributed to the higher diarrhea-related mortality rate among rural children than among their urban counterparts.^23^

The development trend of the driving forces from 2000 to 2020 is illustrated in Figure 2, where the global rural population increased by 9.9%. However, the projected trend for the rural population from 2020 to 2030 fluctuates with an initial decrease followed by an increase. From 2019 to 2023, the rural population declined to the levels observed in 2000 (Figure 2c), likely due to the global COVID-19 pandemic. Additionally, the proportion of the elderly in the population has increased, with an anticipated increase of 4.30% from 2000 to 2030. In contrast, the proportion of children under five years old is expected to decline by 2.60% (Figure 2d).

### 3.2 Changes in DAUSRs

From 2000 to 2030, the annual DAUSRs and mortality rates attributed to unsafe rural sanitation are projected to decrease (Figure 3a), with a reduction of 816.7 thousand and 25.2 per 100,000, respectively. Figure 3(b–e) illustrates the age-standardized death rates attributed to unsafe rural sanitation in global rural areas in 2000, 2010, 2020, and 2030, revealing significant spatial differences in the projected worldwide trends. To illustrate this, we describe the changes in DAUSRs across two types of classifications: the six major geographical regions and the WHO categories (full results are presented in Supporting Information, Figure S2 and S3). From 2000 to 2030, Africa exhibited the highest age-standardized mortality rates attributable to unsafe rural sanitation. The rates decreased from 80.2 (95% CI: 65.0–91.1) to 15.8 (9.2–27.5) per 100,000, a reduction exceeding twice the global average. Despite this significant decline, Africa still has the highest mortality rate worldwide (Figure S2a). In contrast, countries in Southeast Asia saw a decrease in age-standardized death rates from 52.0 to 9.5 per 100,000 over the same 30-year period. The Eastern Mediterranean region started with an age-standardized mortality rate of 32.1 (41.3–57.8) per 100,000 in 2000, which fell below 10 per 100,000 people after two decades. Notably, all regions except Europe showed a downward trend in age-standardized mortality rates, whereas Europe’s rates fluctuated between 0.5 and 1.3 per 100,000 people. It is projected that by 2030, the Western Pacific region will have the lowest age-standardized mortality rates attributable to unsafe rural sanitation globally, decreasing from 2.9 (2.3–3.1) to 0.50 (0.3–0.9) per 100,000 people over a 30-year period. Figure S3a shows that age-standardized death rates decreased from high-income to low-income countries, and except for high-income countries, the age-standardized death rates of other SDI types of countries decrease year by year. During these 20 years, the age-standardized death rates in high-income countries increased by 0.9 (−0.5–23.4) per 100,000 people, echoing the upward trend of European countries. By 2030, the age-standardized deaths rates in low-income countries reached 15.3 (8.5–28.2) per 100,000 people, ten times that of high-income countries. This indicates that low-income countries, especially those in Africa and Southeast Asia, need to improve rural environmental sanitation.

**Figure 3:**
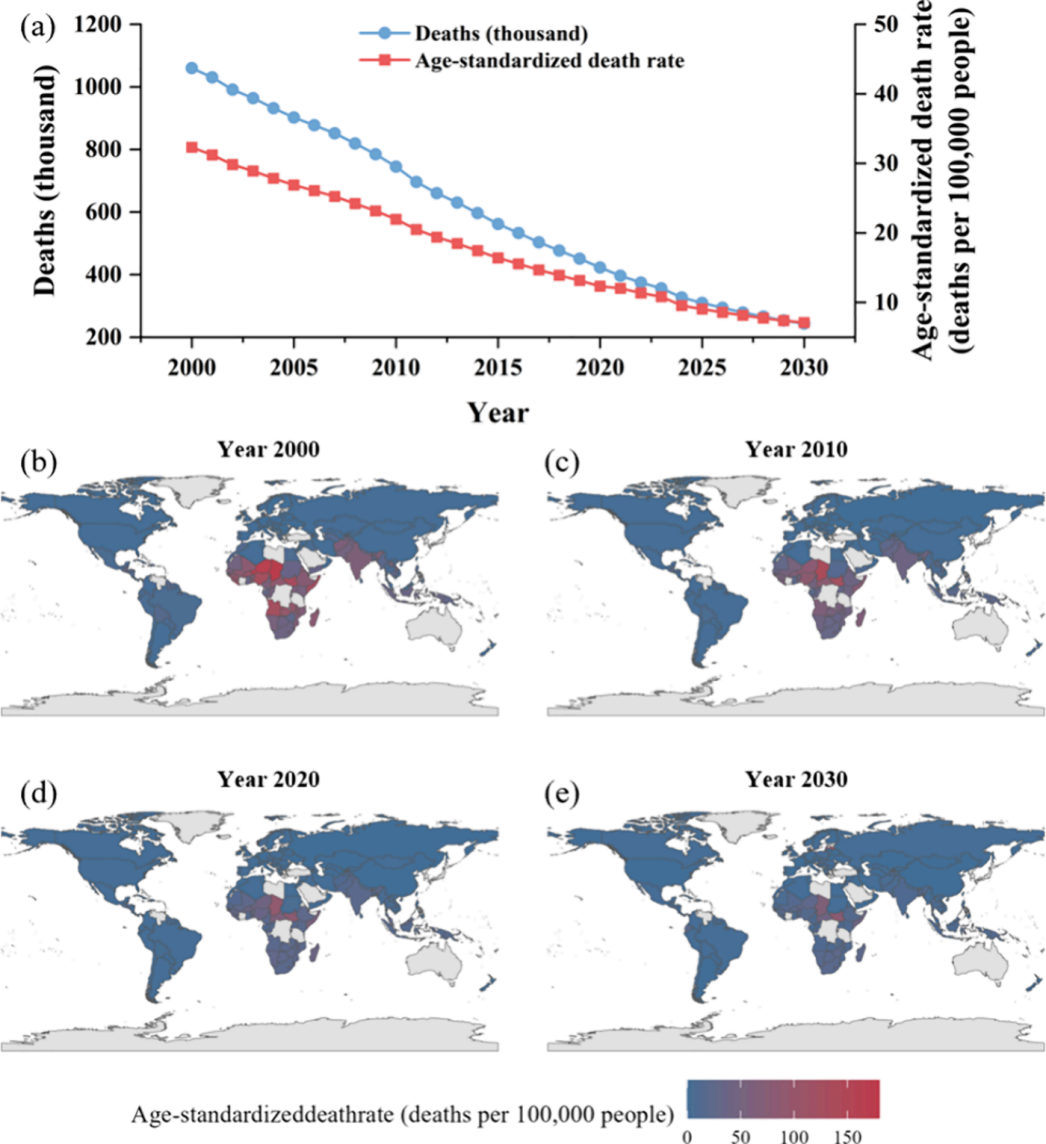
Trends and spatial distribution of global Deaths Attributable to Unsafe Sanitation in Rural areas (DAUSRs) and age-standardized deaths rates from 2000 to 2030. (a) Changes in DAUSRs and age-standardized deaths rates; (b) Spatial distribution of age-standardized deaths rates due to unsafe sanitation conditions in rural areas globally in 2000; (c) Spatial distribution of age-standardized deaths rates due to unsafe sanitation conditions in rural areas globally in 2010; (d) Spatial distribution of age-standardized deaths rates due to unsafe sanitation conditions in rural areas globally in 2020; and (e) Spatial distribution of age-standardized deaths rates due to unsafe sanitation conditions in rural areas globally in 2030.

Figure S2b indicates that Southeast Asia and Africa have the highest death tolls, which are attributed to unsafe rural sanitation. These regions have large rural populations; however, basic environmental sanitation in these rural areas is severely inadequate. The death toll in some countries was significantly higher than the global average. For instance, the estimated death toll of India is considerably higher than that of other countries, reaching 488,073 (388,584–543,287) thousand in 2000, and is projected to decrease by 397,424 (601,09–133,405) thousand in 2030. The high DAUSRs in India were primarily due to its substantial population size.

As shown in Figure 4b, contrary to standardized mortality rates, lower-middle-income countries exhibited the highest DAUSRs, with the largest net reduction over the two decades, dropping from 767,639 (609,485–852,576) to 150,400 (97,007–227,996). By 2030, the DAUSRs of lower-middle- and low-income countries are expected to account for 88% of the global total. This phenomenon is partly because the rural population in lower-middle-income countries constitutes a majority of the global rural population and partly because the environmental and medical conditions in the rural areas of lower-middle-income and low-income countries are relatively poor.^24^ Therefore, low- and middle-low-income countries should draw upon the experiences of developed nations and adapt their schemes to their local contexts to formulate tailored regulations and policies. This approach would improve rural environmental sanitation and healthcare.^25^ High-income countries have achieved a stable phase in the advancement of rural environmental sanitation. Therefore, it is imperative to disseminate successful practices to other regions and establish a resilient management framework for rural environmental sanitation.

**Figure 4:**
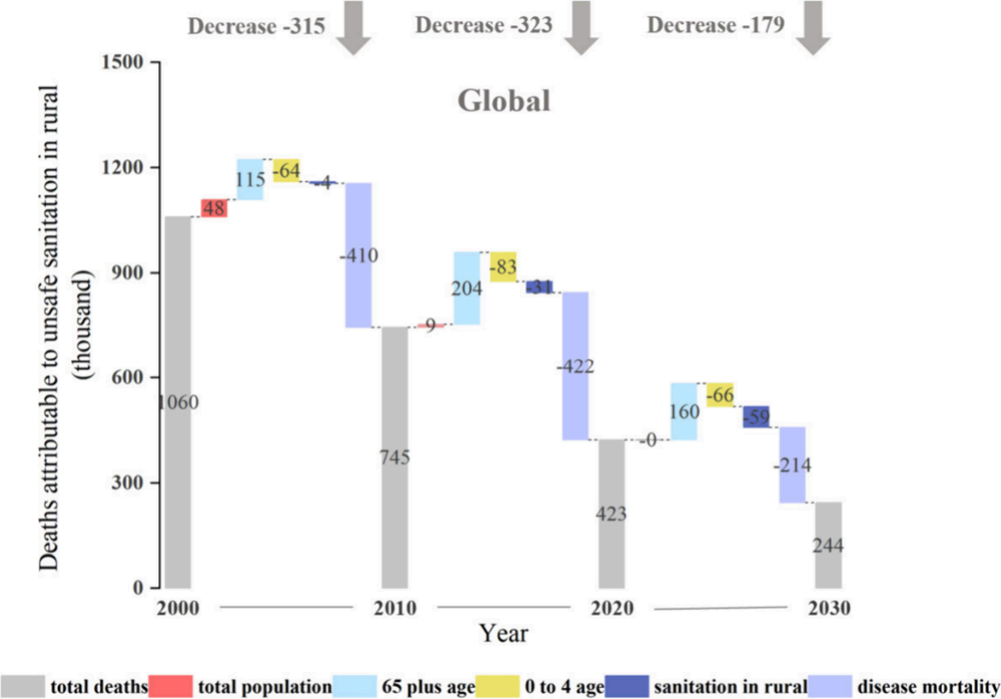
Contributing factors to changes in global deaths attributable to unsafe sanitation in rural areas (DAUSRs) from 2000 to 2030.

Our research indicates that certain regions (such as Europe and other high-income countries) have made positive strides toward both rural environmental sanitation and healthcare. However, there remains significant room for improvement in mitigating the DAUSRs in these areas, particularly given their rapidly aging populations. Conversely, in middle-to-low-income and low-income countries (such as Africa and Southeast Asia), economic and technological constraints continue to expose rural residents to significant environmental sanitation risks. This demonstrates that the challenges of preventing and mitigating DAUSRs vary regionally and represent significant challenges for countries at similar stages of development.

### 3.3 Factors Contributing to DAUSRs

The impact of the five factors on DAUSRs was compared based on the decomposition method (see Materials and Methods 2.3.4 for details). Figure 4 illustrates the contributions of these five factors (total population, proportion of elderly people aged over 65 years, proportion of children aged under 5 years, proportion of sanitary conditions, and disease mortality rates) to the DAUSRs during the periods 2000–2010, 2010–2020, and 2020–2030. Among these factors, rural population aging and population growth corresponded to an increase in 480 thousand and 56.8 thousand deaths, respectively. From 2000 to 2020, global rural aging reached 115 thousand and 204 thousand deaths, respectively. This increase is projected to reduce from 2020–2030, albeit to a small extent. Specifically, the total rural population is gradually decreasing, with population-related DAUSRs decreasing from 47.8 thousand deaths to 9.17 thousand deaths. The proportion of elderly people over 65 years of age is increasing, while the global fertility rate is declining, leading to a reduction in the proportion of the under-4 population, which has somewhat reduced the number of DAUSRs. This is attributed to the high mortality rate caused by diarrhea among infants aged under 4 years, which have a high-incidence rate of the disease.^26^ Reductions in rural populations, fertility rates, and improvements in rural sanitary facilities have slightly reduced the number of DAUSRs. However, more than 85% of this reduction is attributed to improved medical facilities in rural areas.

Declining fertility rates, improved sanitation facilities, and better medical conditions contributed to a decrease of 65.9 million, 59.5 million, and 214 000 deaths, respectively, from 2020 to 2030. Improvements in rural sanitary conditions have significantly contributed to a reduction of 59.5 thousand deaths from 2020 to 2030, marking an additional decrease of 55.3 thousand compared with those from the 2000–2010 period. Additionally, the projected reduction in DAUSRs for the 2020–2030 period is lower than that in the previous phases. This could be due to the diminishing contribution of global medical improvements because most countries have stabilized diarrheal mortality at a relatively low level.^27,28^ For all age groups, in terms of disease mortality, the elderly people aged over 65 years and children aged under 5 years continue to be at risk (Figure S1). By 2030, the mortality rates of diarrhea among the elderly and children are projected to be 318 (167–612) and 138 (14–52) per 100,000 people, respectively, which are significantly higher than those of other age groups.

The contributions of the different drivers to the number of deaths varied globally, as depicted in Figures 5 and S4. For instance, in regions with higher aging rates, such as certain countries in Africa and Southeast Asia, the age structure is the most significant factor affecting changes in the number of deaths. Influenced by an aging rural population, the total number of deaths in these regions continues to increase across all time periods, with an overall increase of approximately 1.2 times over the global average. Considering a country in Africa as an example, the total number of deaths increased by 0.198 thousand between 2020 and 2030. The increase in the total number of deaths was significantly greater than that of the global average. When using the SDI for regional classification, it can be observed that countries in low- and lower-middle-income regions still have significant potential to reduce DAUSRs (Figure S3a and b). Furthermore, in low-income countries, the contributions of various factors during 2000–2010 were markedly different. Relatively poor rural medical conditions were considered the main cause of the DAUSRs, and population decline. Improving rural environmental sanitation still has great potential for reducing DAUSRs in African, South-East Asia, and Eastern Mediterranean regions, where DAUSRs are projected to be 98.2, 119, and 14.8 thousand people, respectively, which will account for 95% of the total deaths in 2030. To maintain a future global reduction in DAUSRs, improving rural medical conditions, advancing rural sanitation toilets, and domestic wastewater collection and treatment are equally important.

**Figure 5:**
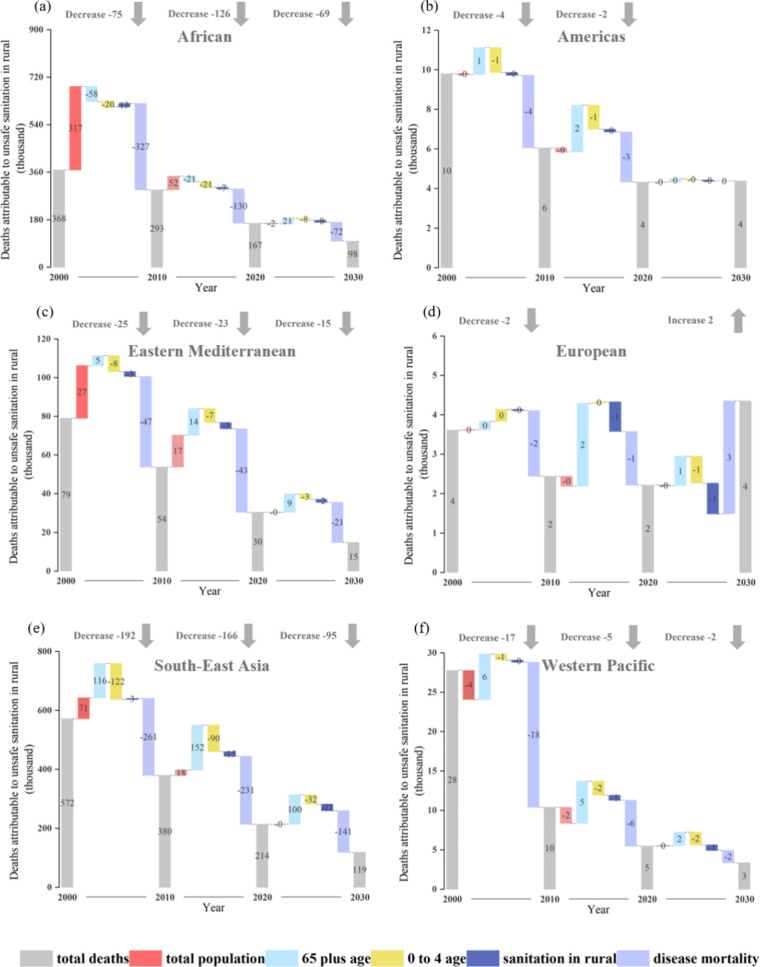
Contributions of various factors to changes in deaths attributable to unsafe sanitation in rural areas (DAUSRs) across six global regions from 2000 to 2030. The figures illustrate (a) Africa; (b) Americas; (c) Eastern Mediterranean; (d) Europe; (e) Southeast Asia; and (f) the Western Pacific, focusing on the cumulative impact of four factors—age structure, total population, air quality, and disease mortality rates—on DAUSRs during the periods 2000–2010, 2010–2020, and 2020–2030.

Overall, the expected rate of DAUSRs mitigation is projected to decrease from 2020 to 2030 (Figure 4). This is primarily attributed to gradual improvements in rural healthcare conditions in most countries, whereas efforts to manage environmental sanitation in rural areas remain relatively slow.^29^ Additionally, aging populations present further challenges in mitigating DAUSRs. The complexity of this challenge is further highlighted by the fact that older individuals are more susceptible to diseases, which could potentially increase DAUSRs, as suggested by Yeatts et al.^30^

### 3.4 Measures and Limitations

This significant and preventable burden underscores the need for countries to prioritize DAUSRs reductions in their public health policies. To alleviate the health burdens caused by unsafe rural sanitary environments and reduce health risks, countries should not only focus on enhancing rural healthcare and environmental sanitation but also pay special attention to vulnerable populations such as the elderly and young children who are exposed to these unsafe conditions. It is recommended that regions at higher risk invest in the construction of indoor sanitary toilets and sewage systems, which would significantly decrease the level of exposure among the elderly and children. For example, the United States implemented the Clean Water Act (USEPA), while China has initiated its “Toilet Revolution”, and Finland, South Korea, Japan, Singapore, and the United States have established dedicated agencies for the management of public restrooms. Additionally, providing information to older adults regarding the use of sanitary toilets and encouraging them to use hygienic toilet practices could further mitigate health risks. Third, governments should support additional investments in healthcare to improve the quality and accessibility. Targeted measures should also be taken to address the medical costs for children and the elderly, thereby reducing their economic burden. By adopting a comprehensive approach that includes improving rural healthcare and environmental sanitation and focusing on vulnerable populations, countries can effectively alleviate the health burden caused by unsafe rural environmental sanitation and promote better public health.

Our study has few inherent sources of uncertainty and limitations. First, we assumed a constant relative risk estimate for the association between unsafe environmental sanitation and diarrheal disease, which may have introduced a degree of error. Second, we lacked historical data on the age structure of rural populations to estimate the health burden of unsafe rural environmental sanitation across various age groups. Consequently, our study postulates that the age structure of rural populations is congruent with that of the overall population, an assumption that may affect our estimations of the DAUSRs. Therefore, the predictions derived from this study only illustrate the long-term health impacts of unsafe rural environmental sanitation. As future research increasingly focuses on environmental sanitation and rural society, such as the relative risk between environmental sanitation and diarrhea, sociologists project a global high-resolution time series of rural population age distribution, which will render the results of this study more precise.

## 4 Conclusions

This study utilized data from the GBD and WHO to estimate global DAUSRs from 2000 to 2030. The analysis delved into changes in DAUSRs and their driving factors by employing a decomposition approach to quantify the relative contributions of population dynamics, sanitation conditions, age structure, and shifts in disease mortality rates. Despite improvements in global rural sanitation, it is projected that by 2030, 12.2% of the rural population will still lack access to sanitary toilets and will persist in open defecation practices, particularly in regions such as Africa and Southeast Asia, where rural inhabitants have a higher mortality rate associated with diarrheal diseases. From 2000 to 2030, the global burden of deaths attributable to unsafe sanitation in rural areas will be expected to decrease. However, mortality rates among the elderly population and children under five years old will remain high, indicating that these groups will continue to face constant significant risks. Aging of the rural population and population growth are major contributors to the increase in the number of deaths, whereas improvements in rural sanitary facilities and medical support will help reduce the death toll, especially in low- and lower-middle-income countries. Comprehensive measures are imperative for effectively mitigating the health burden associated with unsafe rural sanitation. They include the augmentation of rural medical facilities, advancement of sanitation infrastructure, and prioritization of vulnerable groups, such as the elderly and children. Such targeted endeavors are not only essential for national rural public health policies but also provide support for global sustainable development. Future research will focus on evaluating the specific impacts of different interventions and how to tailor these measures to local conditions to more effectively reduce the health burden associated with unsafe rural sanitation.

## Supplementary Material



## Data Availability

The data that support the findings of this study are available from the corresponding author upon reasonable request.

## †

Haidian District, Shuangqing Road, No. 18, Beijing 100085, PR China
