# Supplementary material for: Aging Population and Lacking Sanitation Governance: Global Challenges in Alleviating Deaths from Unsafe Rural Sanitation
Source: Environ Health (Wash). 2025 Feb 28;3(6):626–35. doi: 10.1021/envhealth.4c00246 (PMC12186206; doi:10.1021/envhealth.4c00246)
Supplement: Supplementary file 1 [file eh4c00246_si_001.pdf]

Supporting Information

Aging population and lacking sanitation governance: Global challenges in alleviating deaths from unsafe rural sanitation

Zixuan Wang [abc§], Pengyu Li [abc§], Wenkai Li [d], Yingnan Cao [ae], Jianguo Liu [e], Lin Li [ab], Junxin Liu [abc], Tianlong Zheng [abc*]

a. State Key Laboratory of Environmental Aquatic Chemistry, Research Center for Eco-Environmental Sciences, Chinese Academy of Sciences, Beijing 100085, China

b. University of Chinese Academy of Sciences, Beijing 100049, China

c. National Joint Research Center for Ecological Conservation and High Quality Development of the Yellow River Basin, Beijing 100012, China

d. SCEGC No. 12 Construction Engineering Group Co., Ltd., Ankang National High-Tech Industries Development Zone, Ankang 725000, Shaanxi, China

e. Key Laboratory of Environmental Pollution Control and Remediation at Universities of Inner Mongolia Autonomous Region, College of Resources and Environmental Engineering, Inner Mongolia University of Technology, Hohhot 010051, Inner Mongolia, China

* Email: tlzheng@rcees.ac.cn

§ Zixuan Wang and Pengyu Li contributed equally to this work.

* Tianlong Zheng: Present Addresses: Haidian District, Shuangqing Road, NO. 18, Beijing 100085, PR China.

Contents

Table S1. Model prediction performance evaluation

Fig. S1. Distribution of diarrheal mortality rates among rural residents aged 0 to 65 and older in 2000 and 2030. Blue represents the year 2000, while red represents the year 2030.

Fig. S2. Changes in age-standardized mortality rates and death counts across six major global regions from 2000 to 2030. Changes in age-standardized mortality rates and death counts across six major global regions from 2000 to 2030: (a) Age-standardized deaths rates and (b) Deaths.

Fig. S3. Changes in age-standardized mortality rates and death counts across SDI categories from 2000 to 2030. Changes in age-standardized mortality rates and death counts across six major global regions from 2000 to 2030: (a) Age-standardized deaths rates and (b) Deaths.

Fig. S4. Contributions factors to changes in deaths attributable to unsafe sanitation in rural areas (DAUSRs) across sdi levels from 2000 to 2030. The figures illustrate (a) High income; (b) Upper middle income; (c) Lower-middle income; and (d) Low income regions, focusing on the cumulative impact of four factors—age structure, total population, air quality, and disease mortality rates—on DAUSRs during the periods 2000-2010, 2010-2020, and 2020-2030.

Table S1. Model prediction performance evaluation

Index	MSE	RMSE	MAE	modle
PAF	2.9296	0.0054	0.0048	ARIMA
Diarrheal mortality rates	52956.6722	230.1231	23.3595	BAPC

PAF: population attributable fraction; ARIMA:AutoRegressive Integrated Moving Average; BAPC: Bayesian age-period-cohort

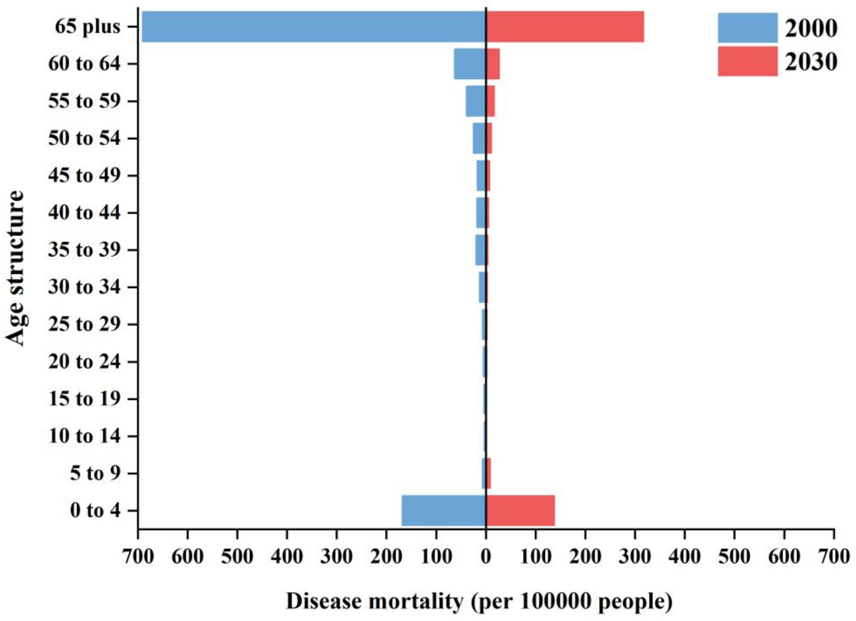

Fig. S1. Distribution of diarrheal mortality rates among rural residents aged 0 to 65 and older in 2000 and 2030. Blue represents the year 2000, while red represents the year 2030.

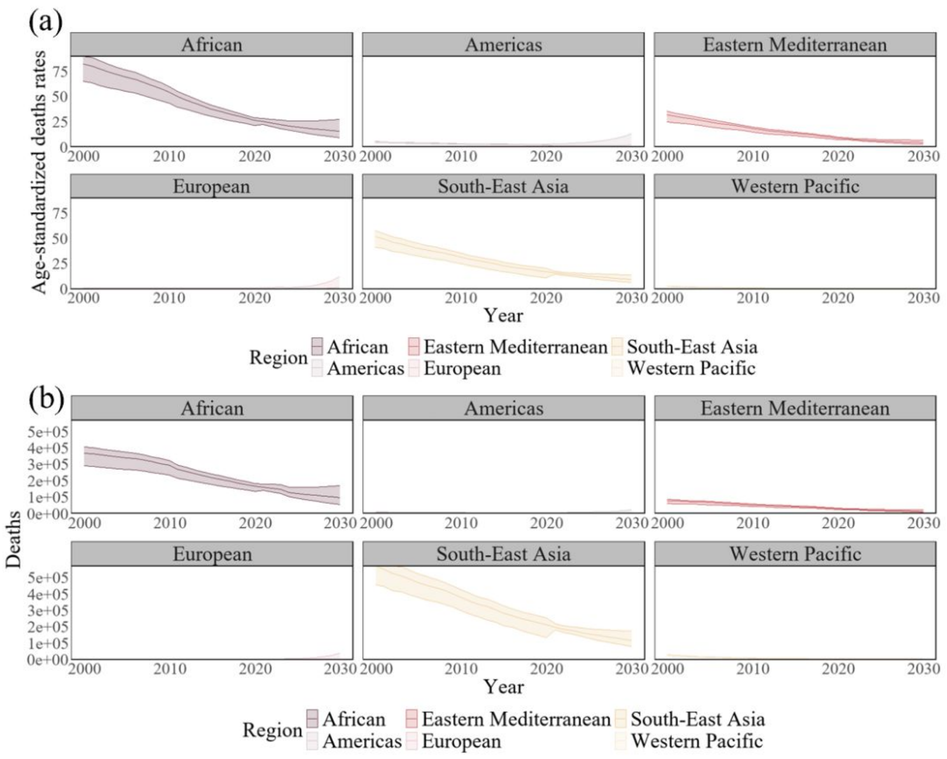

Fig. S2. Changes in age-standardized mortality rates and death counts across six major global regions from 2000 to 2030. Changes in age-standardized mortality rates and death counts across six major global regions from 2000 to 2030: (a) Age-standardized deaths rates and (b) Deaths.

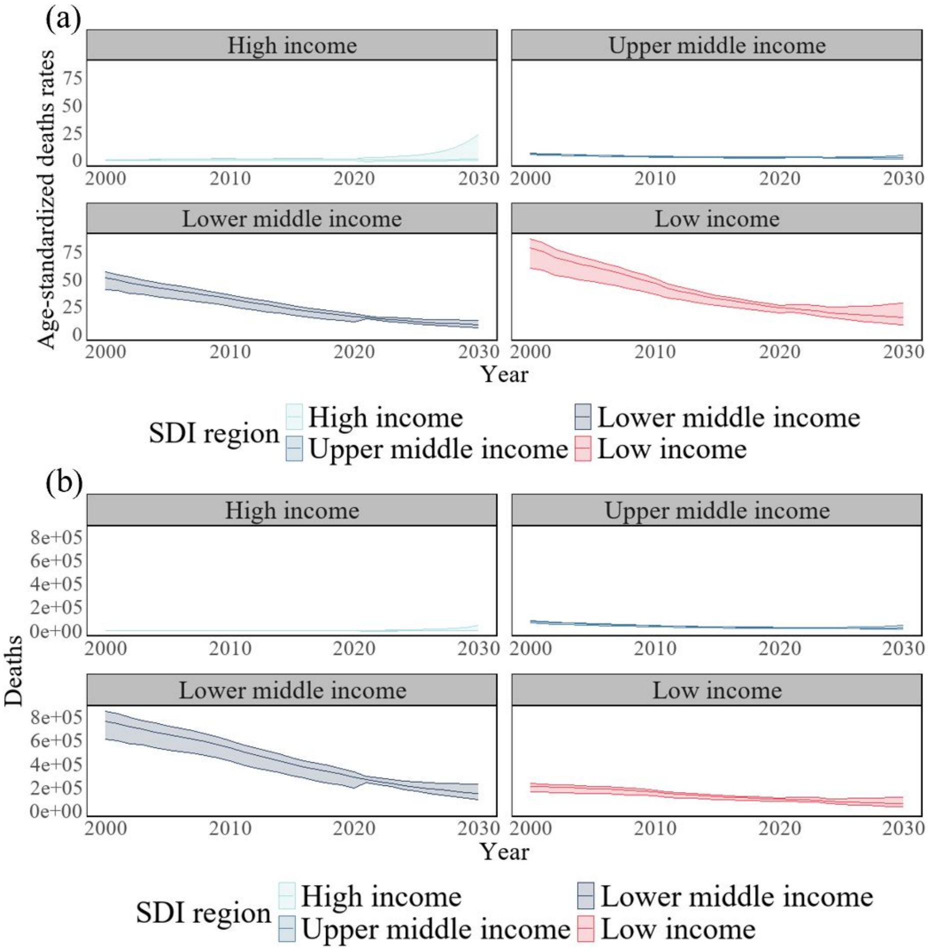

Fig. S3. Changes in age-standardized mortality rates and death counts across SDI categories from 2000 to 2030. Changes in age-standardized mortality rates and death counts across six major global regions from 2000 to 2030: (a) Age-standardized deaths rates and (b) Deaths.

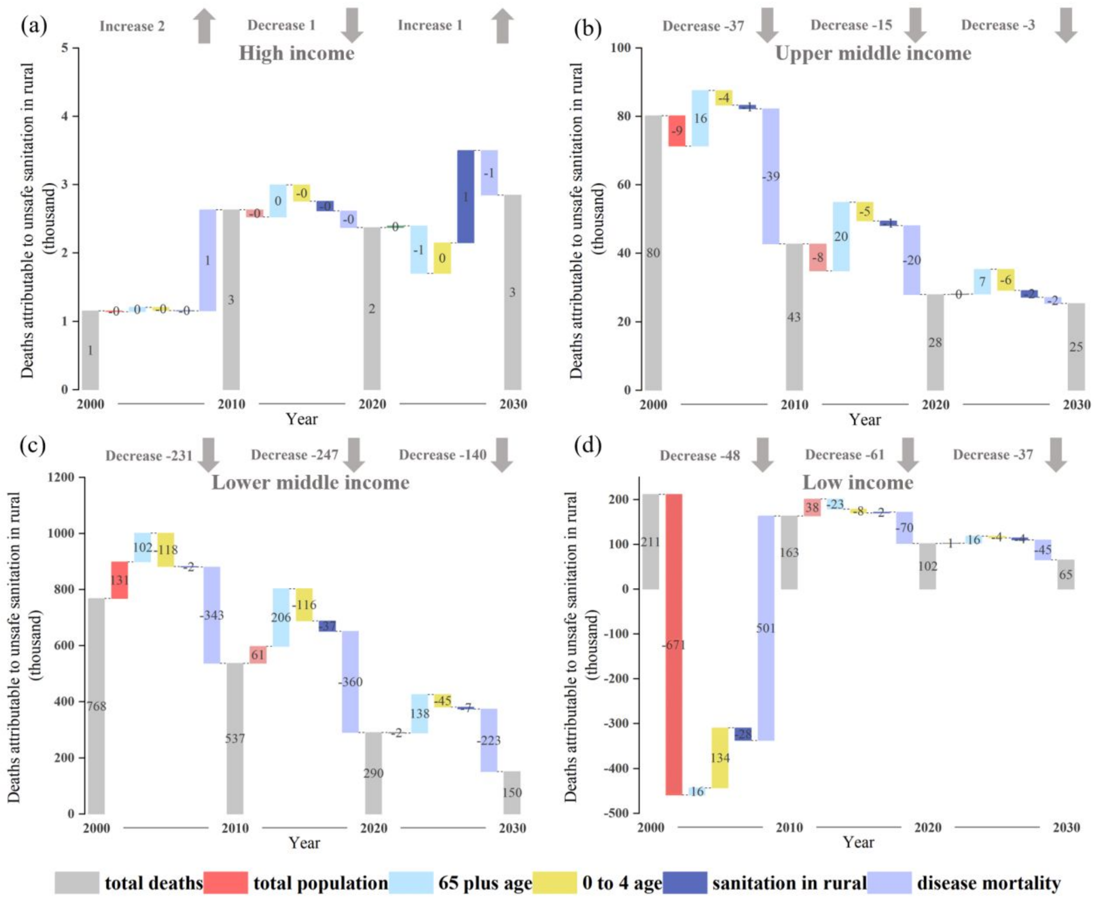

Fig. S4. Contributions factors to changes in deaths attributable to unsafe sanitation in rural areas (DAUSRs) across sdi levels from 2000 to 2030. The figures illustrate (a) High income; (b) Upper middle income; (c) Lower-middle income; and (d) Low income regions, focusing on the cumulative impact of four factors—age structure, total population, air quality, and disease mortality rates—on DAUSRs during the periods 2000-2010, 2010-2020, and 2020-2030.
